# Comparison of Different Bone Filling Materials and Resorbable Membranes by Means of Micro-Tomography. A Preliminary Study in Rabbits

**DOI:** 10.3390/ma12081197

**Published:** 2019-04-12

**Authors:** Enrique Fernández-Bodereau, Guillermo Dedossi, Victor Ortega Asencio, Manuel Fernández-Domínguez, Sérgio Alexandre Gehrke, Juan Manuel Aragoneses, José Luis Calvo-Guirado

**Affiliations:** 1 Department of Prothodontics, Universidad Nacional de Córdoba, Córdoba 5100, Argentine; fernandezenrique5@gmail.com (E.F.-B.); dedosi75@gmail.com (G.D.); 2 Department of Implant Surgery, CEU San Pablo University, 28223 Madrid, Spain; v.ortegasensio@gmail.com (V.O.A.); clinferfun@yahoo.es (M.F.-D.); 3 Department of Research, Biotecnos, Cuareim 1483, CP 11100, Montevideo, Uruguay; sergio.gehrke@hotmail.com.; 4 Department of Dental Research in Universidad Federico Henriquez y Carvajal (UFHEC), Santo Domingo 10107, Dominican Republic; jaragoneses@ufhec.edu.do; 5 Faculty of Health Sciences, Universidad Católica San Antonio de Murcia (UCAM), 30107 Murcia, Spain

**Keywords:** amniotic, collagen, ground teeth, fascia lata, hydroxyapatite, bone, membranes, microCT, bone substitutes

## Abstract

The purpose of this work was to evaluate the behavior of different membranes and bone filling materials used to fill critical defects in rabbit calvaria. Four defects were prepared in the cranial calvaria of female rabbits. They were randomly divided into three subgroups according to the type of barrier membrane to be used. Four animals carried cross-linked bovine collagen membranes (Mem-Lok, Bio-Horizons, Birmingham, AL, USA)), four human fascia lata membranes (Tissue, Inbiomed SA, Córdoba, Argentina) and four human chorioamniotic membranes (Tissue. Inbiomed SA, Córdoba, Argentina). The defects were filled with the deproteinized bovine bone particulate Bio­Oss® (Geistlich­Pharma AG, Wolhusen, Switzerland), with particulate human hydroxyapatite MinerOss® (Bio-Horizons, Birmingham, AL, USA), with particulate dental material (Tissue Bank Foundation, Inbiomed S.A., Córdoba, Argentina), and the last one was left without the addition of filler material. In the first group of four specimens, a resorbable cross-linked bovine collagen membrane was placed over the skull and defects, without additional fixing. In the second group, a human fascia lata membrane was placed, without additional fixing. In the third group, a human chorioamniotic membrane was placed, without additional fixing. The animals were sacrificed at 4 and 8 weeks. The highest percentages of relative radiological density (average) were recorded considering the amnio-chorionic membranes (83.63%) followed by collagen (81.44%) and finally the fascia lata membranes (80.63%), but the differences were not statistically significant (p > 0.05). The sites grafted with a decellularized tooth (96.83%) and Bio­Oss (88.42%), recorded the highest percentages of radiological density but did not differ significantly from each other (subset 2). The three membranes used did not show statistical differences between them, in any of the two time periods used. There were statistical differences between the filling materials evidencing the presence of a large quantity of calcified material in the defects treated with particulate tooth and deproteinized bovine bone and while smaller amounts of calcified material were registered in the case of defects treated with human hydroxyapatite and those that were not treated.

## 1. Introduction

Dental implant placement is often made more difficult by the presence of alveolar ridge defects. Large vestibular cortical bone defects in the ridge can compromise both implant placement and the aesthetics of the subsequent restoration. The increase in osteoclastic activity due to resorption of bone bundle bone during the initial healing phase decreases after 4 to 8 weeks [1], which provides a favorable environment for regeneration procedures, which is mostly necessary in the sites of aesthetic implants to compensate for alterations of the crest in the facial aspect [2,3,4]. A bone replacement material must have a "bimodal" behavior that, in the early stages of differentiation, will allow osteoblasts to build bridges between different grain sizes and integrate with other osteoblasts, supporting both proliferation as the differentiation. The intrinsic stimulation of new bone formation will be supported by the activation and absorption of mesenchymal stem cells on surfaces with nanoscale topographic features [5,6,7].

Many techniques are available for repairing bone defects using xenografts, allografts, alloplastic grafts with or without barrier membranes. Of course, autologous bone grafting remains the gold standard in this type of procedure because of its osteoconductive and osteoinductive properties, and it is biological safety, but it is always associated with some degree of morbidity at the donor site and the quantity of bone available is limited. Dentine and bone have similar components; both are made up of 10% liquid, 20% organic material and 70% minerals, mainly hydroxyapatite, growth factors such as insulin-like growth factors (IGF)-II, as well as transforming growth factors (TGF) and bone morphogenetic proteins. Moreover, cementum contains TGF-I and collagen types I and II [8]. In order to work with a homogenous sample regarding the Ca/P ratio of the bony structures and the type and size of the collagenic fibers, only 10-month-old females that were never fertilized and shared the same type of feeding and captivity were used [9,10,11,12]. The osteoinductive potential was discovered in 1967 and since then various lines of animal research have shown that demineralized dentine matrix induces the formation of ectopic bone in subcutaneous locations and intramuscular pockets [13,14]. However, Kadkhodazadeh et al. (2015) [15] found evidence that fresh autografts of mineralized dentine and cementum had little or no effect on the induction of new bone in post-extraction dental alveoli (in a dog study). The purpose of this work was to evaluate the behavior of different membranes and bone filling materials used to fill critical defects in rabbit calvaria. The behavior of the materials was analyzed by means of high-resolution microtomography.

## 2. Materials and Methods 

**Figure 1:**
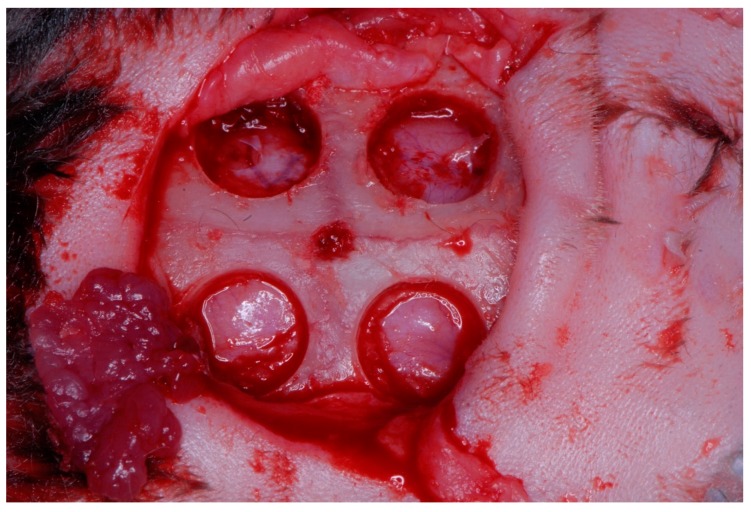
The four different calvarial critical defects after bone removing.

**Figure 2:**
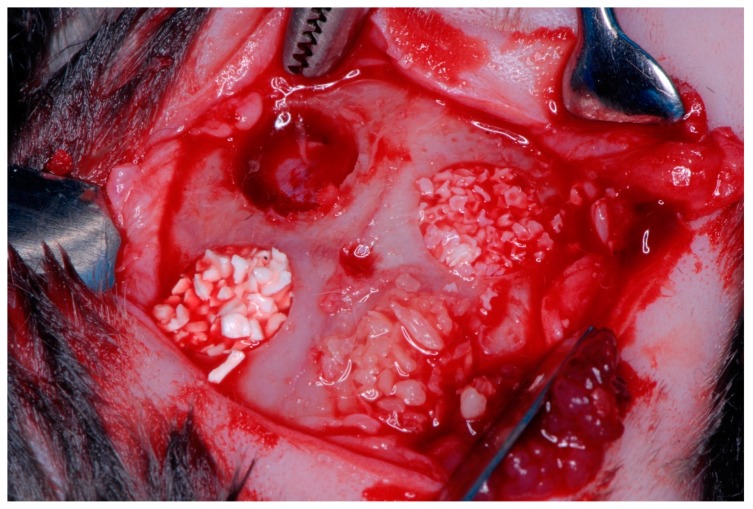
The three different materials and control gap.

**Figure 3:**
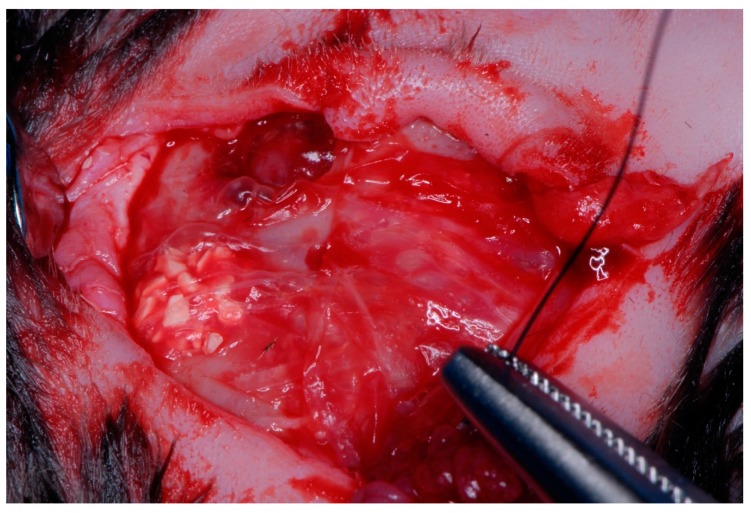
The suture is done in different layers in order to avoid wound opening.

The study was approved by the Ethics and Bio-safety Committee of the Faculty of Veterinary Science, National University of the Littoral (Santa Fe, Argentina) (Protocol Number 148/12) Authorization to conduct the study at the Paraná Agricultural Experiment Station (EEA, Paraná, Argentina) was given by the director of the Entre Rios National Institute of Agricultural Technology (I.N.T.A. Paraná, Central Region, Argentina), which followed guidelines established by the Council Directive of the European Union (53/2013; February 1, 2013) for animal care and experimentation. The study used 12 Female rabbits of the varieties New Zealand meat rabbit/Californian meat rabbit, each with an average weight of 3 kg. The animals were kept in separated cages in a single room and fed a daily diet of pellets and watered ad libitum throughout the study period. The animals were divided into three sub-groups according to the type of barrier membrane applied: Subgroup 1: four specimens received reconstituted cross-linked bovine collagen membranes (Mem-Lok, BioHorizons, Birmingham, AL USA); Subgroup 2: four specimens received human fascia lata membranes (supplied by Biostar-Argentina Foundation Tissue Bank, Córdoba, Argentina); Subgroup 3: four specimens received human chorioamniotic membranes (In-Biomed-Argentina Foundation Tissue Bank) [16,17,18]. Four critical, complete thickness defects of 6-mm diameter were created in the rabbit calvaria using a trephine and surgical guide, two in the frontal area and two in the parietal area, two on the left and two on the right [19,20,21] (Figure 1, Figure 2 and Figure 3). Critical defects generated is one that cannot be repaired at the same time by the specimen in a certain period of time [22]. Preoperatively, the rabbit skulls were shaved and disinfected with SEA 4 (Blue Sea Laboratories, Alicante, Spain). The animal’s heads were first shaved with an electric hair cutter and the skin was disinfected with a povidone-iodine solution (10 g/100 ml). The animals were anesthetized using 0.4 mL Xylazine, 0.3 mL ketamine, and 0.2 mL dexamethasone administered parenterally, with a further 0.2 mL Xylazine injected in the auricular vein, which produced 30–40 min general anesthesia [17]. The animals were pre-anesthetized with 10% zolazepam at 0.10 mL/kg and acepromazine maleate (Calmo-Neosan®, Pfizer, New York, NY, USA 0.12–0.25 mg/kg and medetomidine 35 mg/kg (Medetor 1 mg, Virbac, CP-Pharma Hand-elsgesellschaft GmbH, Burgdorf, Germany). The mixture was injected intramuscularly into the quadriceps femoris. The animals were taken to the operating theatre where, at the first opportunity, an intravenous catheter (diameter 22 G or 20 G) was inserted into the cephalic vein, and propofol infusion was administered at a rate of 0.4 mg/kg/min at a constant infusion rate. Anesthetic maintenance was performed with volatile anesthetics and the animals underwent tracheal intubation with a Magill probe for the adaptation of the anesthetic device and for the administration of volatile isoflurane diluted in oxygen (2 V%). In addition, local anesthesia (Articaine 40 mg, 1% epinephrine, Bernabo, Argentina.) was administered at the surgical sites. These procedures were carried out under the supervision of a veterinary surgeon [23,24].

Surgery commenced with a semi-circular incision made by a nº 15 scalpel, its concavity in a caudal direction, cutting through skin, subcutaneous tissue, and periosteum. The incision extended from a 1 cm caudal of the right superciliary arch to 1 cm caudal of the left superciliary arch. A total thickness flap was lifted and held from the surgical area using a Cawood-Minnesota type retractor. A surgical guide was used to position the defects on the skull cap and a partial thickness perforation was made with a round bur at the conjunction of the sagittal/midline suture and the coronal suture where the surgical guide’s guide pin was inserted. Four defects were created on the exposed cranium with a 6 mm diameter trephine at low speed (150 rpm) and with constant irrigation with physiological saline. Complete trephination perforating the whole bone thickness was not performed, but penetrated the external cortical and spongy bone without cutting into the internal cortical in order to avoid lesioning the dura mater. 

Showing the central position of the Snail1 in the bone remodeling pathways. In wild-type mice, Snail1 is necessary for the first steps of osteoblast differentiation in a dual manner. It activates the expression of the early differentiation markers Collagen I and Osteopontin while it inhibits Runx2 expression, necessary for differentiation to proceed. The transient nature of Snail1 expression allows the second wave of Runx2 transcription needed for bone mineralization. On the other hand, Snail also directly represses Vdr transcription in osteoblasts providing another link between osteoblastogenesis and osteoclastogenesis. Consequently, the sustained Snail1 activation in adult Snail1-ER mice supports the formation of the osteoid but inhibits osteoblast and osteoclast terminal differentiation, leading to a defective mineral deposition.

Snail1 is to be the first transcriptional repressor of Runx2 which, together with the post-translational regulation co-ordinated by Twist and Schnurri-3 [25,26], controls the complex dynamics of Runx2 protein activity during osteoblast differentiation. Snail1 also favors the synthesis of the unmineralized matrix by indirectly activating Collagen I expression and it controls osteoclastogenesis by directly repressing Vdr transcription. Thus, Snail1 lies upstream of three fundamental pathways in bone remodeling and the control of adult bone mass. That aberrant expression of Snail1 would lead to osteomalacia even under normal ion homeostasis conditions as it occurs in the presence of vitamin D and a wild-type Vdr gene.

The internal cortical was finished using an nº 11 scalpel and the (total thickness) bone pieces were detached and removed using an nº 11 scalpel and 3-mm diameter Molt curette. All specimens underwent the same protocol: one defect was filled with deproteinized bovine bone (Bio-Oss, Geistlich-Pharma AG, Switzerland) hydrated with physiological saline; another defect was filled with calcined human bone (human hydroxyapatite) (Miner-Oss, BioHorizons, AL, USA) hydrated with physiological saline; the third defect was filled with ground decellularized, dehydrated dental material (supplied by the Biomed-Argentina Foundation Tissue Bank) hydrated with physiological saline; and the fourth defect was left unfilled by any aggregate material, constituting a negative control group [27,28,29,30]. The main differences between the 3 fillings used are given by their origin, 2 are human (Miner-Oss and tooth material), 1 is calcined hydroxyapatite (Miner-Oss), 1 decellularized and dehydrated (particulate dental material) and 1 bovine bone deproteinized (Bio-Oss).

**Figure 4:**
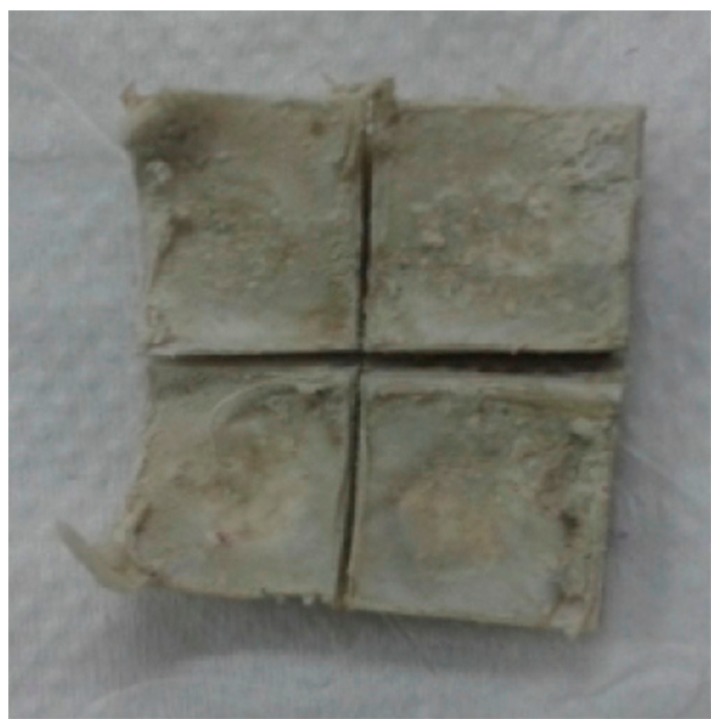
The cabbits calvaria after 8 weeks of healing divided into 4 pieces for histology and CT.

In the first group of four specimens, a resorbable cross-linked bovine collagen membrane was placed over the skull and defects, without additional fixing. In the second group, a human fascia lata membrane was placed, without additional fixing. In the third group, a human chorioamniotic membrane was placed, without additional fixing. The flap was repositioned and sutured with 3.0 nylon making four horizontal double loop mattress sutures, with five simple stitches between them. After surgery, each specimen was administered (intra-muscularly) with a 1 mL dose of penicillin with streptomycin (2.5000.000 U.I.) every 48 h (3 doses), and 2 mL dexamethasone. During the post-operative period, the animals were housed at the I.N.T.A. Paraná Cuniculture Unit in individual cages and fed a balanced diet and water. Four weeks later, half the specimens (n = 6) were sacrificed by concussion and exsanguination [31]. Afterward, the skull cap and periosteum were removed from the animals, making incisions with a cutting disc, labeling the samples, and fixing them in 0.4% formaldehyde. Eight weeks after surgery, the remaining half of the specimens (n = 6) were sacrificed by the same procedure, removing samples, labeling and fixing them as before, for subsequent microtomography analysis (Figure 4).

### 2.1. Microtomography Analysis 

**Figure 5:**
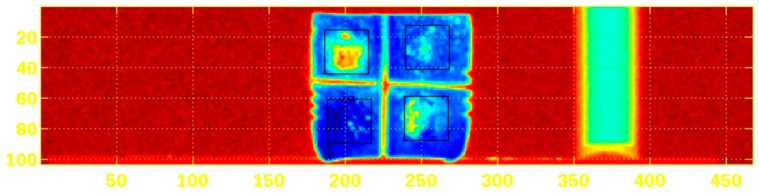
The occlusal view of Micro computerized calvaria after 8 weeks.

Prepared, fixed, and labeled, the samples were sent to the LIIFAMIRx laboratory (Research and Instrumentation Laboratory for Applied Physics in Medicine and X-ray Imaging) at the Faculty of Astronomy, Mathematics, and Physics, the National University of Córdoba (Córdoba, Argentina), where each sample underwent high-resolution microtomography analysis. This used a conventional 3 kW high-stability X-ray tube as a light source (Cristalo Flex, Röntgen Aparat (R), Heinsberg, Germany). A mechanic-electronic device with automatic controls and synchronized multiple degrees of freedom was used for positioning the samples. Direct digital detection was performed with a certified high-resolution, a flat panel detector (Varian Medical Systems, San Francisco, California, USA, model PAX SCAN 2020. Processing and analysis were conducted under a license (Nº 3407-8985-4332-9223-7919) issued by Matlab (The Math Works, (R), Natick, MA, USA), (Figure 5). Sample scanning extended over the entire dimensions of each sample. Two-dimensional radiological image capture was performed in 0.45° steps, obtaining 800 projections per revolution at 2 λ for the complete tomography reconstruction of each sample. Zr and Al filters were used to eliminate low energy photons. A tube containing distilled water was placed beside each sample in order to obtain a known pattern that would make it possible to standardize data obtained from the samples. High resolution computed microtomography is a non-destructive analytic procedure that is able to characterize samples by determining their morphology and analyzing their structural properties. The analysis method consists of determining morphology by means of the physical and spatial properties based on the sample’s absorption/transmission of radiation. The samples’ morphology is obtained from the spatial distribution of electronic density ρe, proportional to energy density ρ, by means of absorption coefficients µabs obtained in measurements of radiation intensity transmitted by the sample (J(x)), relative to the incident (J(x = 0)): in which Δx is the sample thickness and S(E) the spectral characteristics of the radiation beam.

**Figure 6:**
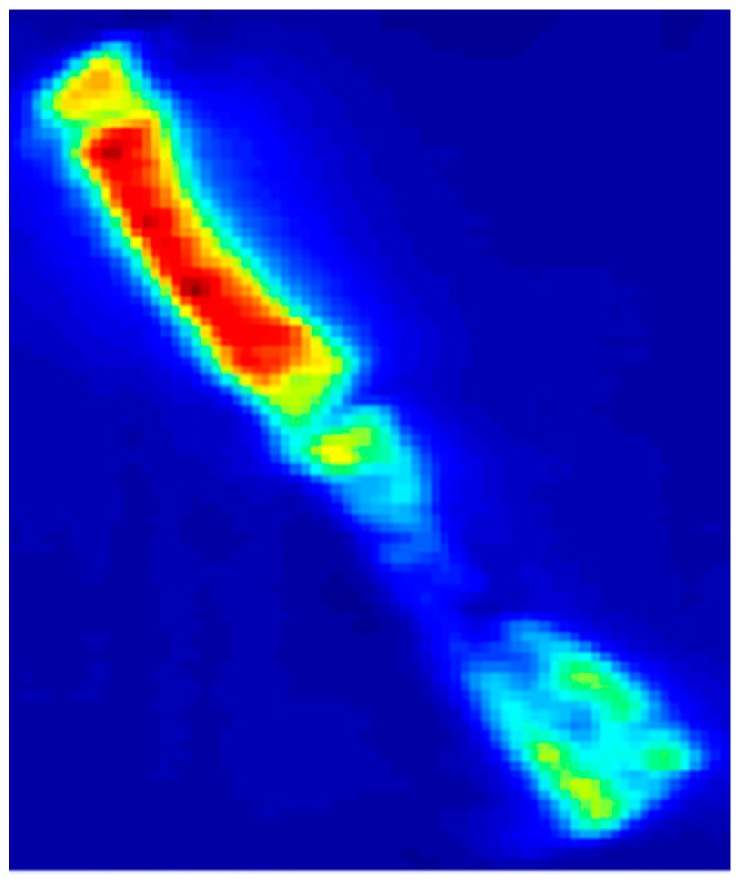
The calvaria lateral view of computerized tomography.

**Figure 7:**
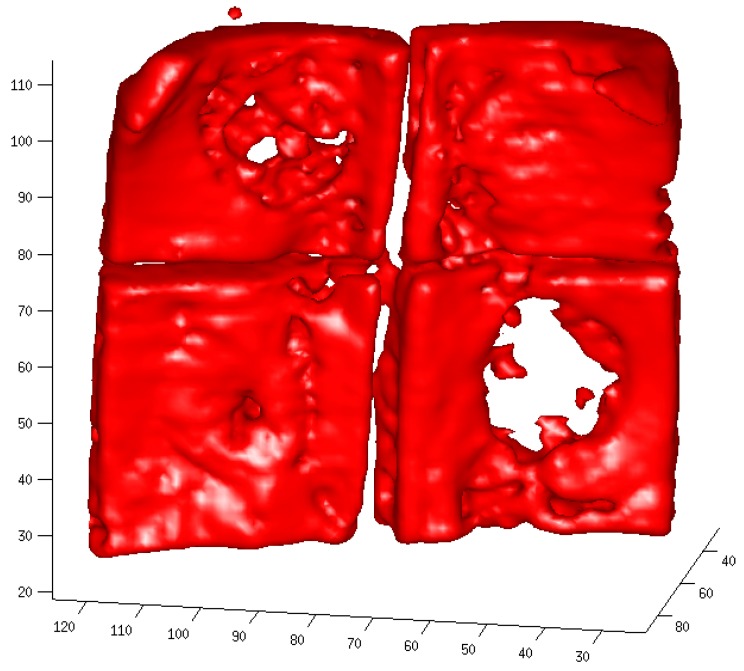
The occlusal view of Micro computerized calvaria at 8 weeks.

Tomography reconstruction completed the analysis process: determining from projections f(ρ, Ø), the spatial distribution of the quantity f (x,y), and so obtaining the sample´s distribution of densities. Energy density ρm is obtained from electronic density ρe, using the X-ray linear attenuation coefficient for energy E that describes the actual fraction of photons interacting per 1-unit thickness of material, which—in the case of the present microtomography technique and its operative regime—is due basically to coherent scattering (to a lesser extent), photoelectric effect and incoherent scattering. It is important to understand that it is relative radiological density that was measured, so that when the filling material in the defect was characterized by greater radio-opacity than the specimen’s native bone, then the filling presented a percentage of radiological density greater than 100% (Figure 6 and Figure 7). 

In this way, radiological density provided an indication of the calcified material present in the defect, although it did not specify whether this consisted of filling material or new bone, or the proportion of one to the other. When three-dimensional tomography reconstruction was complete, the defect areas were defined, in a front view, as an area measuring 29 voxels wide x 28 voxels high x the sample’s bone thickness. A quadrangular area slightly smaller than the defect diameter was selected, as this was a shape that would be easier to repeat than the cylindrical shape of the spatial projection, and so would reduce the scope for error when repeating the model from sample to sample. In each of the 29 slices, an area was defined, 29-voxels long by the projected width of the bone thickness. The radiological density was measured in each of these areas using Image J software version 1.48v; Java 1.6.0.20 (32 bit). The radiological density was also measured in a non-intervened area of bone close to the defect, taking 20 measurements. Ten radiological densities of a standard pattern were also measured (distilled water) in order to standardize all samples. Distilled water did not represent a measurement extreme, but was used as a known pattern to standardize the samples, so that when a standardized divider was applied to each measurement, all environmental variables could be eliminated. So, when the 29 radiological density measures had been obtained from each sample, a mean value was calculated and divided by the mean reading of the standard pattern for each specific sample. In the same way, the mean radiological density of non-intervened bone was divided by the standard pattern mean value for the specific sample. The mean value pertaining to non-intervened bone was defined as 100%, so the relative radiological density for each individual specimen (filled defect) could be calculated as a percentage. 

### 2.2. Statistical Analysis

Data are expressed as mean and standard deviation (SD). The Shapiro–Wilk test was utilized for normality analysis of the parameters. Median, minimum and maximum values of the bone formation measurements were calculated, and the nonparametric Tukey HSD test was used to compare data from micro CT and histomorphometric analysis. The comparison of variables among the 4 groups was performed using the three-way ANOVA model. Pairwise comparisons were performed using the Bonferroni test. All tests were two-sided and statistical significance was set at p < 0.05.

## 3. Results

The radiological density percentages registered at the two study times (4 and 8 weeks) according to the membrane type and graft material are shown in Table 1. 

Multifactorial analysis (three-way ANOVA) was carried out in order to evaluate the influence of variables—membrane type, defect filling material, and time—on the percentage of radiological density in defects, and possible interactions between the variables (Table 2). 

According to this analysis of the different factors evaluated, only the type of filling material proved to be a factor significantly affecting the percentage of radiological density (p < 0.05). The variables’ membrane type, and time, or interactions between variables were not found to influence the radiological density percentages (p > 0.05). The particulate dental filling (96.83%) and Bio-Oss (Geistlich-Pharma AG, Wolfhusen, Switzerland) (88.42%) obtained very high radiological density values, but without significant differences between them (constituting a subset: Subset 2). Miner-Oss. (BioHorizons, Birmingham, AL USA) obtained low percentages of radiological density (76%), with values only slightly higher than those of the unfilled defects (70.33%), without significant difference between these two groups (Subset 1). It should be noted that the materials in Subset 1 showed statistically significant differences in comparison with Subset 2 (Table 3 and Table 4). 

As shown in Table 5, the material obtaining the highest radiological density values at both study times—and so associated with greater efficacy—was the dental filling material, followed by Bio-Oss. The differences between materials were more pronounced at the first study time (after four weeks) and tended to diverge after 8 weeks, but nevertheless maintained the same order of efficacy.

Taking membrane type into account, the highest (mean) percentages of radiological density were registered with chorioamniotic membranes (83.63%) followed by collagen membranes (81.44%), and lastly fascia lata membranes (80.63%), although the differences between membranes were not statistically significant (p > 0.05), making it possible to group all membranes in a single homogenous subset. (Table 6 and Table 7).

**Figure 8:**
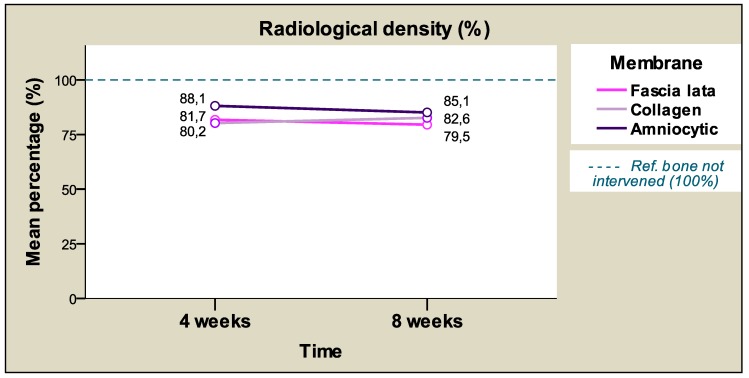
The relative radiological density percentages (average) according to the membrane and time.

**Figure 9:**
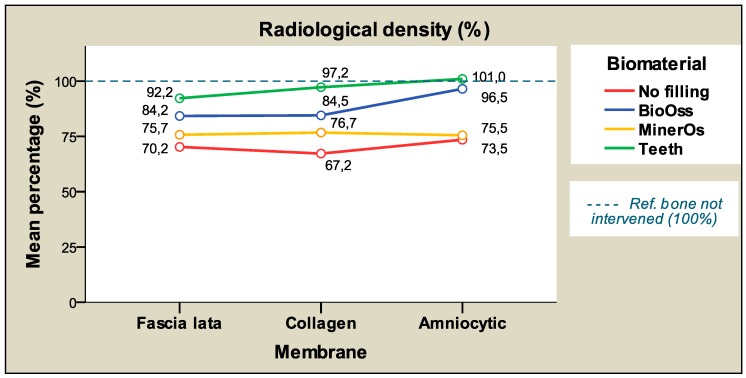
The radiological density percentages (average) according to filling material and membrane.

Table 8 and Figure 8 show the mean radiological density values according to the membrane type and time, presenting similar values at both times for each membrane. The results show less disparate values at the later study time (8 weeks), indicated by the standard deviations (SD) calculated. The differences between study times were not significant. Figure 9 shows the mean radiological density percentages in terms of filling material and membrane that are very similar for each material regardless of the membrane applied.

**Figure 10:**
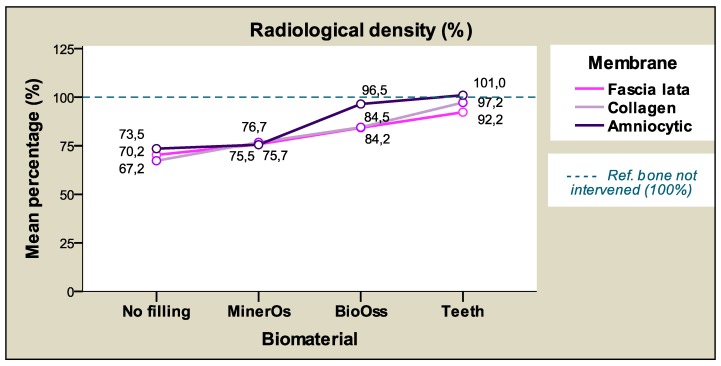
The radiological density percentages (average) according to the membrane and filling material.

This corroborates and provides a graphic representation of the non-interaction between these variables. Figure 10 is a representation of the same data from a different perspective, showing the differences between filling materials and the only slight differences between membranes.

### Histological Section

Each tissue specimen was fixed in a formalin solution (neutral buffered, Sigma-Aldrich Co. LLC., St. Louis, MO, USA) for 2 weeks and then dehydrated while increasing the ethanol concentration of the tissue specimen. All the samples were dehydrated in a graded ethanol series (70, 90, 95% and absolute ethanol) after which the specimens were infiltrated with Technovit 7200 VLC, light curing resin (Heraeus Kulzer, Hanau, Germany) for 30 days while the percentage of resin was increased. Then, all the samples were embedded and light polymerized with an EXAKT 520 polymerizator system (EXAKT Technologies, Oklahoma City, OK, USA) and the resin was solidified for 1 day. Coronal sections were cut using the EXAKT 310 CP cutting unit (EXAKT Technologies, Oklahoma City, OK, USA). The sections obtained were approximately 250 µm in thickness and were manually polished to a final thickness of 50–60 µm; These tissue specimens were dyed with hematoxylin and eosin and then sealed. Histological evaluation was carried out using a light microscope (BX51; Olympus, Tokyo, Japan) coupled to an image analysis system (i-Solution; technology, Daejeon, Korea) under 100× magnification. Images were captured using a digital camera (CC-12; Soft Imaging System GmbH, Munster, Germany) attached to the microscope and were displayed on a computer monitor.

**Figure 11:**
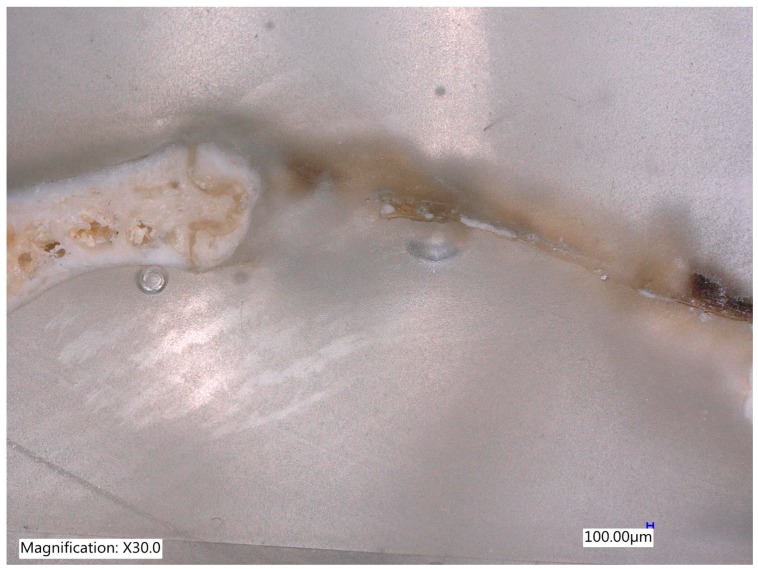
The control site after an 8-week of follow up with complete healing without bone formation (Magnification 30×).

**Figure 12:**
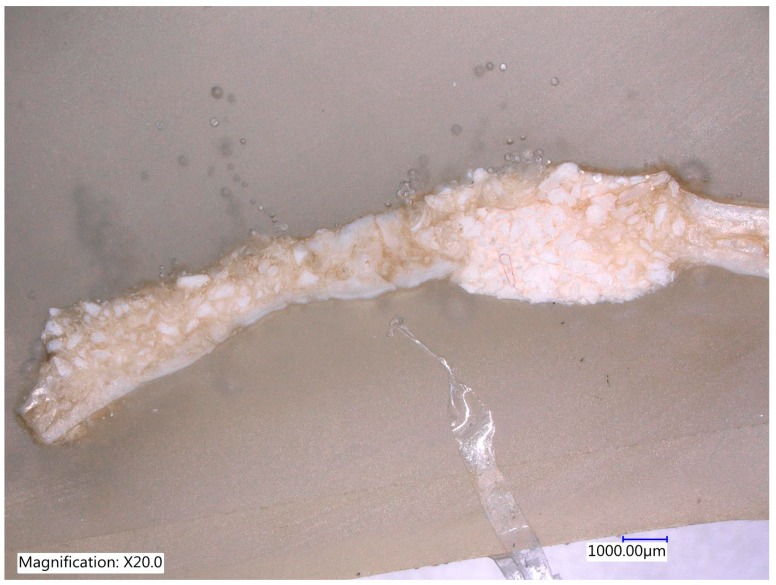
The MinerOs site after an 8-week of follow up with the new bone formation on the right side and on the left side some reduction of biomaterial was observed (Magnification 20×).

**Figure 13:**
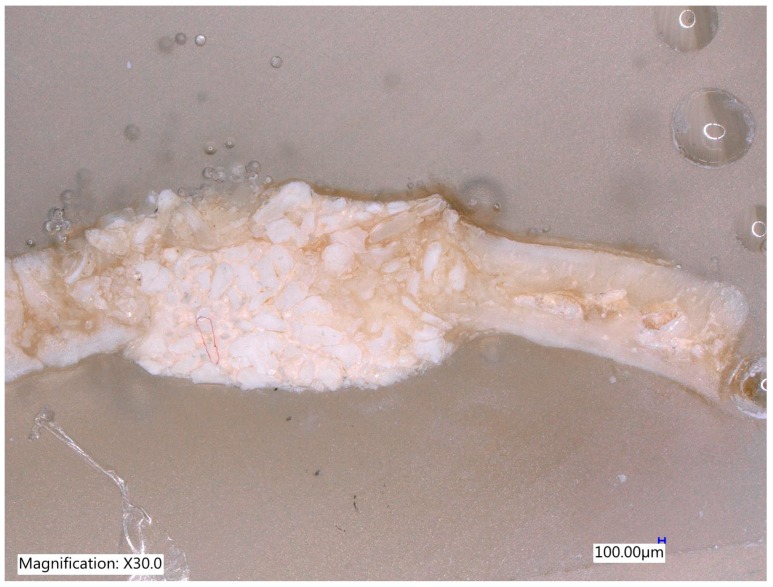
The BioOss site after an 8-week of follow up with the highest new bone formation with new bone in between the particles made the bone more stable (Magnification 30×).

**Figure 14:**
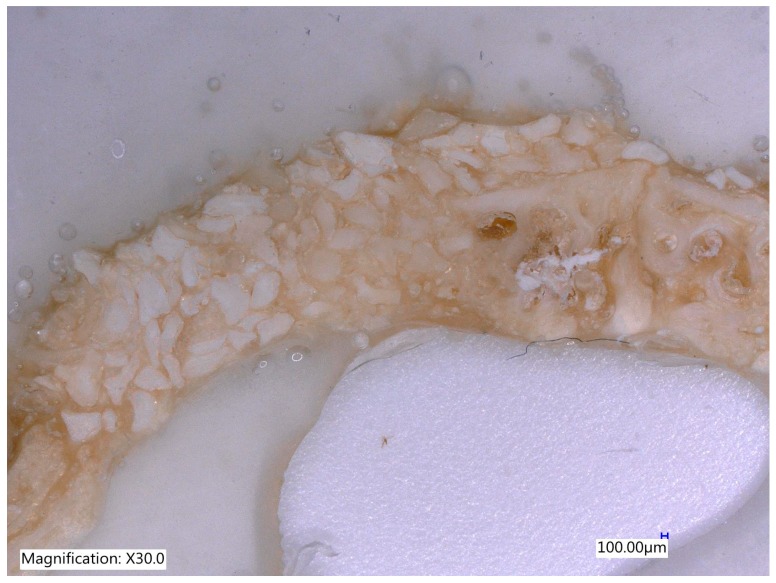
Teeth particles after 8 weeks of evaluation. The original volume of the defects in all specimens was preserved by means of bone graft materials (magnification 30×).

One section from each defect was examined under Digital Microscope Keyence VHX-6000 series (Keyence Corporation of América, 500 Park Boulevard, Suite 200, Itasca, IL 60143, USA) at lower magnification (30×) without staining (Figure 11, Figure 12, Figure 13 and Figure 14). 

The histological images of the four groups showed that within the defect, MinerOs, BioOss and teeth granules of various sizes were homogeneously distributed in the operated area in all skulls. At 8 weeks, the MinerOss group maintained its bone volume relative to 4 weeks, the BioOss group showed significant bone growth and graft resorption, mainly Teeth group, comparable with the rest. The Teeth group also presented low graft resorption by 8 weeks; however, a slight increase in bone volume was observed.

**Figure 15:**
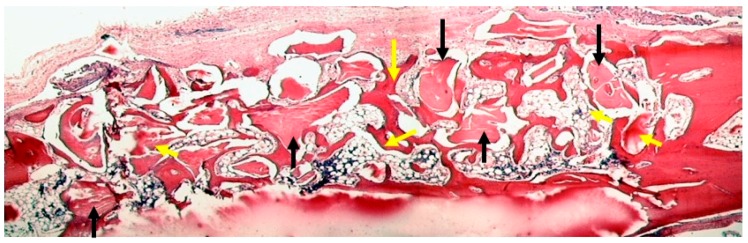
The defects were filled with newly formed bone in areas close to the margin of the defect and in the central defect area in the teeth group at 8 weeks of follow up. Hematoxylin-Eosin staining (Magnification 2×). Black arrows represent teeth particles and yellow arrows described the newly formed bone.

In all groups, new bone formation was increased at 8 weeks of healing when compared with the 4-week evaluation. In areas close to the margin of the defect, the amount of newly formed bone toward the central defect area was increased and grafted particles were in direct contact with newly formed bone tissue in areas showing new bone formation (Figure 15).

## 4. Discussion

The present study identified non-calcified material in higher percentages in the control group (without aggregate filling) than in the groups of filled defects, a finding that concurs with Calvo Guirado et al. [32] who compared hydroxyapatite/β-Tricalcium phosphate (HA-β-TCP) alone, HA-β-TCP with silica aggregate, and unfilled defects, in critical defects in rabbit calvaria, finding a significantly higher quantity of connective tissue in the unfilled control group than the groups with filled defects, regardless of which filling aggregates were used. It is important not to lose sight of the fact that we are measuring the radiological density and not percentage of bone regeneration which implies and explains that when using a filler material that is more radiopaque than native bone, the value of the radiological density is higher than that of native bone and, with the passage of time and product of the degradation-reabsorption of the aggregate material, the values diminish approaching the value of the native bone. In the same way, the defects of the control group (without added filler material) present very low values of radiological density at 4 weeks, increasing at 8 weeks, which is compatible with the normal healing process for the animal model used [15]. Turri et al. [33] described that the combination of a barrier membrane and different bone substitute materials has been shown, both experimentally and clinically, to possess a synergic activity leading to predictable bone regeneration and long-term success, particularly in the field of implant dentistry. This has been backed by numerous studies including Dahlin et al. [34]; Hämmerle and Jung [35]; Stavropoulos et al. [36]; Simion et al. [37]; Donos et al. [38]; Esposito et al. [39]; Jung et al. [40]; Tal ert al. [41].

Aloise et al. [42] compared a xenograft scaffold enriched (EX) with bone marrow mesenchymal stem cells (BM-MSCs), un-enriched xenograft alone (UX) and autogenous bone graft (ABG) with and without collagen membranes to fill defects in rabbit calvaria. They found that the use of collagen membranes produced a synergic effect in the ABG and UX groups but not in the EX group; it would appear that the use of the membrane made no difference in the presence of pluripotent bone marrow cells. According to Martinez et al. [43], the action of a membrane as a bone growth enhancer was demonstrated by the complete closure of the bone defect at unfilled control sites (in a study comparing deproteinized bovine bone with β-tricalcium phosphate used to fill rabbit calvaria defects), even though there was a partial collapse of the membrane. Hammerle and Jung [35] induced bone regeneration in the space under the membrane, and for some authors, the presence of the membrane is the principal element on which bone defect healing depends. Donos et al. [44].

The present study found no significant differences between the different membranes used. But significant differences were found between the types of filling materials used, which could be grouped as two subsets according to the degree of radiological density achieved. One subset was made up of particulate dental filling material and deproteinized bovine bone (Bio-Oss), while the other subset consisted of calcined human bone (Miner-Oss) and blood coagulate without aggregate material (unfilled control defects). It is clear from the present results that the highest radiological density values were obtained by the particulate dental filler, like this a material with a higher initial level of (mineralization) than the native host bone and has particles that resorb slowly. So, the lowest radiological density values were seen with the blood coagulate that entered unfilled defects following the normal healing pattern for the species, particularly at the 4 weeks study time.

Following the results of Pang et al. [45], where he used a matrix of autogenous demineralized dentin from the extracted teeth in the extraction cavities of the graft and increasing the vertical dimension; it was found that this was as effective as the increase of using an organic bovine bone.

Despite the rapid metabolic activity of the New Zealand rabbit, other studies have established their validity as an experimental model to test the biomaterials used for bone replacement (Ramírez-Fernández et al., 2011) [46]. In view of this, the results in this study were improved by the creation of critical defects that were 6 mm in diameter, which will not close spontaneously and, therefore, will demonstrate the regenerative potential of the biomaterials under observation [47].

Hammerle et al. [35]: defects filled with human hydroxyapatite and deproteinized bovine bone produced significant differences, an unexpected finding that could not be explained by microtomography findings and will be investigated through histomorphometric analysis at a later stage. As argued by Martínez et al. [43], it is likely that the nature of the graft material plays a relevant role in the quantity of bone formation. The reabsorption of bone graft materials has presented a problem to both researchers and clinicians [48]. For many years it was thought that the embryonic origin of a graft material played an important role in its reabsorption capacity and that those of a membranous origin were able to resist reabsorption better than those of endochondral origin [49,50]. However, more recent research has revealed that the proportion of cortical to trabecular bone is more important than the grafts’ embryonic origin [51,52]. Likewise, it is interesting to remember that the degree of compaction of the graft material has a direct relationship with the degree of resorption of the same, as established by Delgado Diaz et al. [53] and in the present study and because it was a critical bone defect of the total thickness, the degree of compaction was minimal, given that the pressure exerted would have sent the graft material to the inside of the skull of the specimens. Moreover, the porosity and surface irregularity of a graft material’s particles play a fundamental role in the reabsorption time [54].

## 5. Conclusions

The three membranes compared did not show statistically significant differences between them at either study time (4 weeks or 8 weeks). Statistically significant differences were found between the filling materials used, with the presence of a large quantity of calcified material in defects treated with particulate dental filling and deproteinized bovine bone, and smaller quantities of calcified material in defects treated with human hydroxyapatite and defects not filled with an aggregate material.

## Figures and Tables

**Table 1:** The relative radiological density percentages recorded at 4 and 8 weeks according to the membrane type and graft filling (red tones express low percentages of radiological density and green tones high percentages).

Time	Membrane	Biomaterial			
		No filling	MinerOs	BioOss	Teeth
4 weeks	Fascia Lata	57%	73%	83%	99%
		70%	76%	93%	103%
	Collagen	57%	77%	87%	108%
	Mem-block	66%	77%	82%	88%
	Amniocytic	78%	57%	99%	79%
		73%	85%	107%	127%
8 weeks	Fascia Lata	75%	74%	71%	77%
		79%	80%	90%	90%
	Collagen	75%	85%	93%	100%
	Mem-block	71%	68%	76%	93%
	Amniocytic	69%	83%	92%	91%
		74%	77%	88%	107%

**Table 2:** Multifactorial ANOVA: Factors; Sum of squares (SS); Degrees of freedom (df); Quadratic mean (QM); Fisher’s statistical (F) and statistical significance (p-value). Significant value p < 0.05*.

Factors	SS	df	QM	F	p-value
Material	5161.23	3	1720.41	14.920	*<0.001*
Membrane	339.04	2	169.52	1.470	*0.250*
Time	11.02	1	11.02	0.096	*0.760*
Material * Membrane	288.96	6	48.16	0.418	*0.860*
Material * Time	492.73	3	164.24	1.424	*0.260*
Membrane * Time	67.79	2	33.89	0.294	*0.748*
R square = 0.709 (R square corrected = 0.431)					

**Table 3:** Tukey’s HSD test. Based on the means observed according to the filling material. (*) The difference in means is significant at the 0.05 level.

Biomaterials		Mean Differences	p-value
No filling	MinerOss	−5.67	0.576
	BioOss	(*)−18.08	0.002
	Teeth	(*)−26.50	<0.001
MinerOss	BioOss	(*)−12.42	0.043
	Teeth	(*)−20.83	<0.001
BioOss	Teeth	−8.42	0.247

**Table 4:** Tukey HSD test. The means of percentages differences for the groups in homogeneous subsets are shown.

Biomaterial	n	Groups	
		1	2
No filling	12	70.33	-
MinerOss	12	76.00	-
BioOss	12	-	88.42
Teeth	12	-	96.83
p-value	-	0.576	0.247

**Table 5:** The descriptive statistical values of differences in radiological density percentages: Case count (n); Mean (%) and standard deviation (SD) according to material and stage.

Biomaterial	Time	n	Mean	SD
No filling	4 weeks	6	66.83	8.57
	8 weeks	6	73.83	3.49
	Total	12	70.33	7.23
MinerOss	4 weeks	6	74.17	9.30
	8 weeks	6	77.83	6.24
	Total	12	76.00	7.79
BioOss	4 weeks	6	91.83	9.81
	8 weeks	6	85.00	9.21
	Total	12	88.42	9.75
Teeth	4 weeks	6	100.67	16.65
	8 weeks	6	93.00	10.14
	Total	12	96.83	13.74
Total	4 weeks	24	83.38	17.50
	8 weeks	24	82.42	10.37
	Total	48	82.90	14.24

**Table 6:** Tukey’s HSD test. Based on the means of radiological density percentages observed according to the membrane. The difference between the membranes was not significant.

Membranes		Mean Differences	p-value
Fascia Lata	Collagen	−0.81	0.975
	Amniocytic	−6.00	0.273
Collagen	Amniocytic	−5.19	0.374

**Table 7:** Tukey HSD test. The means of differences in radiological density percentages for the groups in homogeneous subsets are shown.

Membrane	n	Groups
		1
Fascia Lata	16	80.63
Collagen	16	81.44
Amniocytic	16	86.63
p-value	-	0.273

**Table 8:** The descriptive statistical values of differences in radiological density percentages: Case count (n); Mean (%) and standard deviation (SD) according to the membrane and stage.

Membrane	Time	n	Mean	SD
Fascia Lata	4 weeks	8	81.75	15.76
	8 weeks	8	79.50	7.07
	Total	16	80.63	11.85
Collagen	4 weeks	8	80.25	15.32
	8 weeks	8	82.63	11.79
	Total	16	81.44	13.27
Amniocytic	4 weeks	8	88.13	21.97
	8 weeks	8	85.13	12.09
	Total	16	86.63	17.20
Total	4 weeks	24	83.38	17.49
	8 weeks	24	82.42	10.37
	Total	48	82.90	14.24
